# Insights from dual-platform metabolomics on durian flowers: An alternative source of procyanidins from agricultural waste with bioactivities^☆^

**DOI:** 10.1016/j.fochx.2025.102719

**Published:** 2025-07-01

**Authors:** Supakorn Potijun, Nattaya Pattarapipatkul, Pitchakorn Boonma, Putthamas Pewlong, Intira Pathtubtim, Thanchanok Muangman, Bunyarit Meksiriporn, Hubert Schaller, Supaart Sirikantaramas

**Affiliations:** a Program in Biotechnology, Faculty of Science, Chulalongkorn University, Bangkok 10330, Thailand; b Center of Excellence in Molecular Crop, Department of Biochemistry, Faculty of Science, Chulalongkorn University, Bangkok 10330, Thailand; c Institut de biologie moléculaire des plantes, CNRS, Université de Strasbourg, Strasbourg 67084, France; d Expert Centre of Innovative Herbal Products, Thailand Institute of Scientific and Technological Research, Khlong Luang, Pathum Thani 12120, Thailand; e Department of Biology, School of Science, King Mongkut's Institute of Technology Ladkrabang, Bangkok 10520, Thailand

**Keywords:** Anti-inflammatory, Antioxidant, Durian, Green chemistry, Metabolomics, Sustainability, Waste management

## Abstract

Procyanidins, which are polyphenol compounds in grape seeds, apples, and berries, are known for their anti-inflammatory and antioxidant properties. In this study, we investigated durian flowers, an agricultural waste, as a novel procyanidin source. Durian trees bloom prolifically, but not all flowers develop into mature fruits, representing underutilized resources. Dual-platform metabolomic analysis using ultra-high-performance liquid chromatography with electrospray ionization quadrupole time-of-flight mass spectrometry and gas chromatography–mass spectrometry annotates polyphenols such as (−)-epicatechin, procyanidins B1, B2, and C1. The 80 % (*v*/v) ethanol extraction yielded a crude extract with a total procyanidin content of 7.68 mg/g. Bioactivity assays revealed that the procyanidin-rich crude extract reduced oxidative stress and exhibited anti-inflammatory effects against UVA in human keratinocytes (HaCaT). This study is the first to propose durian flowers as a sustainable and cost-effective procyanidin source with potential application in the nutraceutical and cosmeceutical industries, contributing significantly to the repurposing of agricultural waste through green technology.

## Highlights

• Durian flowers are alternative procyanidin sources.
• Durian flower extract has antioxidant and anti-inflammatory effects in HaCaT cells.
• Procyanidin crude extracts exhibit a potential for various applications.
• Durian flower valorization promotes sustainability and reduces agricultural waste.

## 1 Introduction

Climate change is intensifying worldwide, significantly affecting agriculture and human life. Rising global temperatures are disrupting agricultural productivity, and heightened exposure to ultraviolet radiation (UVR) and high temperatures increases health risks. In addition, PM2.5 and PM10 pollution has significantly worsened throughout South, East, and Southeast Asia. Consequently, there is growing interest in sustainable agricultural practices that minimize waste by using all plant components, which could help mitigate the costs associated with climate change. Recent studies have focused on converting agricultural by-products into biofuels and nutraceuticals, with the latter gaining attention due to their high value and environmental benefits (Singh et al., 2020). However, developing nutraceuticals for human supplements is a lengthy process. In contrast, cosmeceuticals can be developed more rapidly and offer a promising solution to prevent skin diseases, including cancer, particularly as the incidence of skin cancer rises due to environmental pollutants and increased UVR exposure (Parker, 2020). This perspective highlights the potential applications of cosmeceuticals and may serve as a foundation for their development as functional ingredients.

Polyphenols are a group of bioactive compounds known for their potent antioxidant and anti-inflammatory properties as well as other health benefits. These compounds have been widely studied for their potential to protect human skin from environmental stressors (Moreno-Montoro et al., 2015). Among polyphenols, procyanidins are oligomeric compounds consisting of (+) catechin and (−) epicatechin (EP). These naturally abundant compounds are commonly found in vegetables, fruits, legumes, cereals, and seeds (Nallathambi et al., 2020). Grapes, particularly their peels and seeds, are the primary commercial source of procyanidins and contribute significantly to global procyanidin production (Zraik & Hess-Busch, 2021). The procyanidin content in grape seeds varies depending on the grape cultivar and the extraction method used. Among the methods, supercritical CO₂ extraction yields the highest amount of procyanidins, followed by methanol, ethanol, and water extractions. However, despite its efficiency, supercritical extraction involves significantly higher operational costs compared to solvent-based methods (Ghafoor et al., 2012). The reported total proanthocyanidin content reaches up to 35.3 mg/g of seed dry weight, consisting primarily of monomers, such as (+) catechin and (−) epicatechin, and polymers, including dimers, trimers, or larger oligomers of (+) catechins and (−) epicatechins) (Moreno-Montoro et al., 2015). These compounds have antioxidant and anti-inflammatory properties. The potential of grape-derived procyanidins to mitigate the adverse effects of chemotherapy further underscores their therapeutic applications (Olaku et al., 2015). Despite their promising benefits, procyanidin production remains inadequate to meet global demands, and the high cost of grape-derived products limits their accessibility, particularly in tropical regions.

**Fig. 1:**
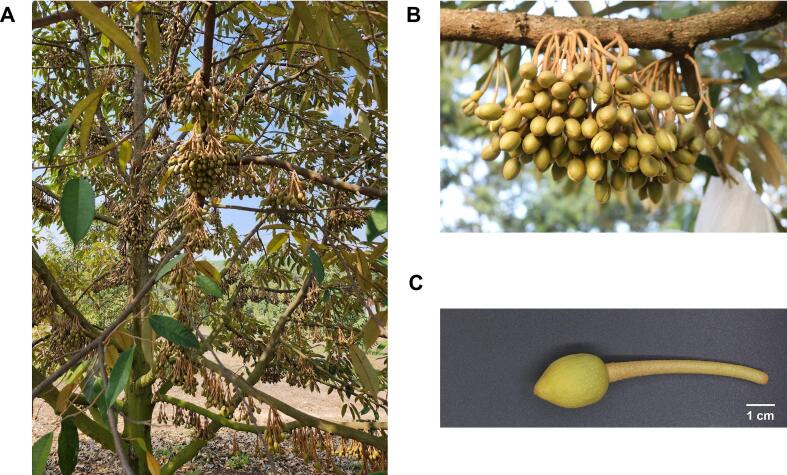
Flowers of the Mon Thong durian cultivar. (A) Durian tree during the flowering stage. (B) Durian flowers at the “Hua Kamrai” (bracelet head) stage before thinning. (C) Durian flower morphology.

Durian (*Durio zibethinus*), known as the “king of fruits” in Southeast Asia, is widely cultivated in Thailand, particularly in the eastern and southern regions (Khaksar et al., 2024). Thailand and Malaysia account for 90 % of global durian production, with Thailand producing 2,435,390 metric tons (t) and Malaysia 448,000 t (Gamay et al., 2024). Durian cultivation has undergone significant expansion, leading to increased agricultural waste (e.g., shells, husks, seeds, and flowers) amounting to over 1 million tons in Thailand in 2020 (Khaksar et al., 2022). Previous studies have reported on the reuse of durian agricultural waste, particularly seeds and husk, for bioethanol, phenolic compounds, and biofilm production (Gamay et al., 2024). However, no previous report has described the use of durian flowers, highlighting a research gap in exploring its potential applications. Durian flowers are classified as agricultural waste because farmers frequently remove excess durian flowers at the bud stage to optimize fruit production (Fig. 1A and B) approximately 60 days before pollination. This practice is widespread in Thailand and leads to the discarding of >80 % of the flowers. A small fraction of these flowers is used as food in some regions (e.g., Thailand, Malaysia, and Indonesia); however, the majority remain unutilized. These discarded flowers are often burned or left to decompose, contributing to greenhouse gas emissions and increasing the risk of fungal infections, particularly those caused by *Phytophthora* sp., thereby severely affecting durian crops (Mahathaninwong et al., 2022).

Our previous study identified early immature durian as a source of (−)-epicatechin and procyanidins (Pewlong et al., 2025); however, the exploration of metabolites in durian residues (e.g., seeds, peels, and flowers) remains limited. In the present study, we investigated durian flowers as an alternative, abundant (−)-epicatechin and procyanidin source. We investigated the bioactive potential of procyanidins derived from durian flowers, focusing on their application as cosmeceuticals for skin cancer prevention, particularly in regions experiencing increased UVR due to climate change. Procyanidins exhibit superior free-radical scavenging activities, and procyanidin-containing lotions and sunscreens exhibit lower ultraviolet transmittance than other polyphenol-based products (Zhou et al., 2021).

In this study, we considered durian flowers as a novel agricultural waste product and assessed their bioactive properties using dual-platform metabolomics. Our analysis revealed that durian flowers are rich in polyphenols, particularly (−)-epicatechin and procyanidins, including, procyanidin B1, B2, and C1. Our quantitative analysis by high-performance liquid chromatography (HPLC) confirmed that durian flowers contain these compounds at concentrations comparable to those found in grape seeds. Our study describes for the first time that durian flowers are rich and sustainable sources of procyanidin, with promising bioactivity, including antioxidant and anti-inflammatory effects in human keratinocytes (HaCaT). Our results indicate that durian flower extracts could be used to develop cosmeceuticals and nutraceuticals. This approach aligns with sustainable management practices by incorporating zero-waste and green chemistry principles, offering an effective strategy to combat environmental stressors and climate change effects on skin health.

## 2 Materials and methods

### 2.1 Plant materials and chemical reagents

Durian flowers (*Durio zibethinus* cv. Mon Thong) were harvested in December 2022 from orchards in Chanthaburi Province, Thailand. The flowers were collected 45–55 days post-flowering at the bud stage and are locally referred to as “Hua Kamrai” (bracelet head) (Fig. 1C). Both the flower heads and stalks were used for further analyses. To ensure biochemical integrity, flower were immediately immersed in liquid nitrogen upon collection and stored at −80 °C until analysis.

The chemicals used in this study included standards such as (−)-epicatechin (EP) and procyanidins B1 (B1), B2 (B2), and C1 (C1) (sourced from ChemFaces, Wuhan, China); D-sorbitol-^13^C_6_ (BLD Pharma, Shanghai, China); (1*S*)-camphor-10-sulfonic acid, formic acid, methoxyamine hydrochloride, *N*-trimethylsilyl-*N*-methyl trifluoroacetamide (MSTFA), puerarin, pyridine, sodium hydrogen phosphate, and sodium dihydrogen phosphate (Sigma-Aldrich, Munich, Germany). Solvents such as acetonitrile, dimethyl sulfoxide (DMSO), ethanol, and methanol were procured from Thermo Fisher Scientific (Seoul, Korea), and the Cellular ROS/Superoxide Detection Assay (ab139476), Human TNF alpha ELISA (ab181421), and Human IL-6 ELISA (ab178013) kits were procured from Abcam (Cambridge, United Kingdom).

### 2.2 Polar phase extraction for dual-platform metabolomics

We followed the protocol outlined in our previous study (Pewlong et al., 2025), with some modifications. The harvested flowers were freeze-dried using a lyophilizer (Buchi, Flawil, Switzerland) for 48 h and ground into a fine powder using a mixer mill (Retsch, Haan, Germany) set to 30 Hz for two 45-s cycles. The extraction was performed by adding 1 mL of 50 % methanol (1:1 methanol:water (%*v*/v)) to 10 mg of the powdered flowers. The internal standards, including D-sorbitol-^13^C_6_ for GC–MS analysis and puerarin and (1*S*)-camphor-10-sulfonic acid for ultrahigh-performance liquid chromatography coupled with electrospray ionization quadrupole time-of-flight mass spectrometry (UHPLC–ESI–QTOF–MS/MS), were dissolved in methanol to a final concentration of 10 ppm before supplementation. The mixture was vortexed for 30 s and incubated on a shaker at 1000 rpm for 1 h at 4 °C using a ThermoMixer C device (Eppendorf, Hamburg, Germany).

Phase separation was performed by adding 1 mL of chloroform, followed by vortexing the sample for 45 s and centrifuging it at 12,000 rpm for 10 min at 4 °C. After centrifugation, the sample separated into two distinct phases, with residues and the polar phase forming the upper layer. A 200-μL aliquot of the polar phase was filtered through a 0.22-μm nylon filter membrane and stored at −80 °C for further analyses.

### 2.3 Dual-platform metabolomics

For the GC–MS analysis, we dried a 200-μL aliquot in section 2.2 using a vacuum concentrator set at 37 °C (CentriVap Benchtop, Labconco, MO, USA). The metabolite sample was derivatized according to the manufacturer's protocol. The dried sample was mixed with 40 μL of methoxyamine hydrochloride (20 mg/mL in pyridine) and incubated at 37 °C with shaking at 1000 rpm for 2 h. Next, 70 μL of MSTFA was added to the mixture and incubated for an additional 30 min at 37 °C. The derivatized sample was then transferred to a gas chromatography vial for analysis. The analysis was performed using a GCMS-QP2020Plus system (Shimadzu, Kyoto, Japan) equipped with an SH-Rxi-5Sil MS column (30 m × 0.25 mm I.D., 0.25 μm df; Agilent Technologies, Santa Clara, California, USA). The injection port temperature was set to 250 °C. The column temperature program was started at 80 °C, held for 1 min, and increased at a rate of 20 °C/min to a final temperature of 300 °C, which was maintained for 45 min. Helium was used as the carrier gas at a flow rate of 1.0 mL/min. The ion source and quadrupole temperatures were maintained at 230 °C and 150 °C, respectively. A 1-μL aliquot of the derivatized sample was injected in the splitless and split modes (20:1), and Peak annotation was performed using spectral matching and chromatographic characteristics, supported by retention indices, as adapted from Lisec et al. (2006). The results are expressed as peak areas normalized to those of the internal standard, D-sorbitol-^13^C_6_.

For the LC–MS/MS analysis, a 200-μL aliquot of the polar phase sample was prepared with puerarin and (1*S*)-camphor-10-sulfonic acid as internal standards. The analysis was performed using UHPLC–ESI–QTOF–MS/MS (Model LCMS 9030, Shimadzu, Kyoto, Japan) in negative ionization mode. A 1-μL aliquot of the sample with internal standards was injected into the UHPLC–ESI–QTOF–MS/MS system and loaded onto a Luna C18(2)-HST column (100 × 2 mm × 2.5 μm; Phenomenex, Torrance, California, USA). The separation and mass spectrometry conditions were based on the protocol outlined by Hutasingh et al. (2023).

### 2.4 Metabolome data analysis

GC–MS data analysis involved meticulous examination of sample chromatograms using LabSolutions GCMS software (version 4.54, Shimadzu, Kyoto, Japan). Derivatized primary metabolites were preliminarily annotated by rigorously comparing their mass spectra against the NIST 20 database, with ≥85 % spectral coverage. This initial process, leveraging unique spectral and chromatographic characteristics, was further supported by calculated retention indices (RIs) using n-alkanes under identical conditions (Table S1). While not definitive for confirmation due to variations from reported values, the consistency of these RIs provided valuable supplementary information, subtly enhancing annotation reliability beyond mass spectral data.

For the LC–MS/MS data analysis, the raw data files were converted into .mzML format using LabSolutions software. The converted data were then processed using the MS-DIAL software (RIKEN, version 5), which facilitated tasks such as peak alignment, deconvolution, feature extraction, normalization, and annotation. For annotation, the MS-DIAL metabolomics MSP spectral kit (2024) was used, which included libraries with EI-MS, MS/MS, and CCS (collision cross-section) values.

### 2.5 HPLC quantification of (−)-epicatechin and procyanidins

The (−)-epicatechin as well as procyanidin B1, B2, and C1 concentrations in durian flowers were determined using HPLC. The mobile phase and elution parameters were optimized based on the method described by Cheevarungnapakul et al. (2019). In brief, 10 mg of powdered durian flowers was combined with 1 mL of 50 %–80 % *v*/v ethanol, with puerarin used as the internal standard. The mixture was vortexed for 30 s and incubated on a shaker at 1000 rpm for 1 h at 4 °C using a ThermoMixer C device (Eppendorf, Hamburg, Germany). After incubation, the mixture was centrifuged at 12,000 rpm for 10 min at 4 °C. A 200-μL aliquot of the supernatant was then filtered through a 0.22-μm nylon filter membrane and stored at −80 °C for subsequent HPLC analysis. HPLC analysis was performed using an Ultimate-3000 HPLC system (Thermo Scientific, Waltham, MA, USA) equipped with a Kinetex C18 reverse-phase column (250 × 4.6 mm, 5 μm; Phenomenex, Torrance, California, USA). Chromatographic separation was achieved using a mobile phase consisting of solvents A (0.1 % trifluoroacetic acid-containing water) and B (0.1 % trifluoroacetic acid-containing acetonitrile) delivered at a flow rate of 1 mL/min. We identified (−)-epicatechin and procyanidin compounds based on the retention times and ultraviolet spectra of authentic standards and quantified their concentrations.

### 2.6 Antioxidant, Total phenolic compound, and procyanidin extraction and quantitative analysis

We extracted freeze-dried durian flower samples with varying ethanol concentrations (50 %–100 %) as described in Section 2.2. The antioxidant capacities of the extracts were assessed using FRAP and Folin–Ciocalteu (FC) assays, as previously described by Boutennoun et al. (2017) for both.

For crude extract preparation, the powdered freeze-dried durian flower sample was extracted with 80 % ethanol for 1 h in an incubator shaker (New Brunswick Scientific, Edison, New Jersey, USA) at 250 rpm and incubated at 25 °C in a conical flask. The supernatant was then collected via centrifugation and filtration using a 0.45-μm nylon membrane filter. The filtrate was concentrated using a rotary evaporator (Buchi, Flawil, Switzerland) at 35 °C and subsequently converted to powder via freeze-drying.

We determined the (−)-epicatechin, B1, B2, and C1 concentrations in the powdered crude extract before storage at −20 °C in the dark. For quantitative analysis of (−)-epicatechin and procyanidin content, the crude extract powder was dissolved in 80 % ethanol at a concentration of 1 mg/mL and analyzed using the HPLC methods described in Section 2.5.

### 2.7 MTT assay-based cell culture and cytotoxicity evaluation

We cultured the human immortalized keratinocyte cell line (HaCaT), obtained from ATCC (Virginia, USA), in Dulbecco's modified Eagle's medium (DMEM) supplemented with 2-mM GlutaMAX, 10 % fetal bovine serum (FBS), and 1 % penicillin-streptomycin. All reagents were sourced from Gibco/Invitrogen (New York, USA). The cells were maintained at 37 °C in a humidified atmosphere with 5 % CO_2_. For subculturing, adherent cells were detached using 0.25 % trypsin-EDTA and passaged every 3 days.

The cytotoxicity of the crude extract was assessed using HaCaT cells seeded at a density of 10^4^ cells per well in a 96-well plate. Next, the cells were treated with varying concentrations of the crude extract (0–2000 μg/mL). After the incubation period, cell viability was determined using the MTT assay, as described by Van Meerloo et al. (2011), with some modifications. Briefly, the cells were incubated with 1 mg/mL of MTT solution at 37 °C for 4 h. After incubation, the medium was replaced with 100 μL of DMSO to solubilize the formazan crystals. The absorbance was measured at 540 nm using a microplate reader (BioTek, Vermont, USA), and cell viability was expressed as a percentage relative to the untreated control.

### 2.8 Assessment of intracellular antioxidant and anti-inflammatory activity in UVA-induced HaCaT cells

HaCaT cells were seeded at a density of 10^6^ cells/well in a 12-well plate and cultured in DMEM supplemented with 2-mM GlutaMAX, 10 % FBS, and 1 % penicillin-streptomycin for 24 h at 37 °C in a 5 % CO_2_ incubator. The following day, the cells were irradiated with UVA at 7 J/cm^2^ using a 1000-W xenon lamp equipped with a dichroic mirror (Oriel, Paris, France). Following UVA exposure, cells were treated with durian flower crude extract at concentrations of 125 and 250 μg/mL, with procyanidin B2 (20 μM) as a positive control. Intracellular reactive oxygen species (ROS) (total ROS and superoxide) levels were measured using the Cellular ROS/Superoxide Detection Assay Kit following the manufacturer's protocol. The extracts were dissolved in DMSO and applied to HaCaT cells as described. The cells were treated simultaneously with the crude extracts of durian flower (125 and 250 μg/mL), procyanidin B2 (a positive control), pyocyanin (an ROS inducer), *N*-acetyl-L-cysteine (a negative control and an ROS inhibitor), and vehicle (untreated samples). After a 30-min incubation at room temperature, ROS induction was initiated by adding 0.1 mL of ROS/Superoxide Detection Solution (2×), containing the vehicle, durian flower crude extract, and ROS inducer. Next, the samples were incubated at 37 °C in the dark for 30 min to 1 h. After incubation, the cells were analyzed using a microplate reader (BioTek, Vermont, USA), measuring the fluorescence at excitation/emission wavelengths of 490/525 nm. Each treatment was performed in duplicate, and the experiments were repeated independently at least three times.

Culture media collected from HaCaT cells were used after the intracellular ROS measurement experiment for the anti-inflammatory activity assay. The collected media were centrifuged at 3000 rpm and 4 °C for 10 min to remove debris. The supernatants were assayed directly or diluted to 1:10 in Sample Diluent NS, followed by storage at −20 °C. Before the assay, all reagents and samples were equilibrated at room temperature. For the TNF-α- and IL-6 measurements, the Human TNF alpha ELISA and Human IL-6 ELISA Kits were used following the manufacturer's instructions. The microplate wells were loaded with 50 μL of durian flower crude extract and procyanidin B2, followed by 50 μL of the antibody cocktail. The plate was incubated for 1 h at room temperature on an incubator shaker at 400 rpm. After incubation, the wells were washed three times with 350 μL of Wash Buffer PT. Next, 100 μL of TMB Development Solution was added, and the mixture was incubated for 10 min in the dark. The reaction was quenched with 100 μL of stop solution, and the absorbance of the wells was read at 450 nm. Alternatively, TMB development can be kinetically monitored in real-time immediately after reagent addition. The treatments were performed in duplicate, independently repeating each experiment at least three times.

The antioxidant and anti-inflammatory activity inhibition percentages (%I) of the sample were calculated based on its activity compared with the control:

$$I=\left(\left[C-S\right]/C\right)\times 100,$$

where I, C, and S represent percent inhibition, control activity (i.e., the activity in the control group), and sample activity (i.e., the activity in the experimental group), respectively.

### 2.9 Statistical analysis

Data from triplicate biological analyses are presented as the mean ± standard deviation (SD). One-way analysis of variance (ANOVA) followed by Tukey's post hoc test was used to determine differences between mean values. We considered *P*-values <0.05 as statistically significant. Data were statistically evaluated using GraphPad Prism 8 software (GraphPad Software Inc., San Diego, CA, USA).

## 3 Results and discussion

### 3.1 Dual-platform Metabolomic analysis of durian flower

We performed dual-platform metabolomic analysis, including GC–MS and UHPLC–ESI–QTOF–MS/MS, to profile and annotate the metabolites. The metabolite profiles observed in this study (Table 1) were similar to those reported in early immature durian in our previous study (Pewlong et al., 2025). Our GC–MS analysis focused on low-molecular-weight metabolites (<400 Da), enabling the annotation of 13 primary metabolite derivatives as trimethylsilyl (TMS) derivatives (Table S1). These metabolites were divided into three major categories: sugars (sucrose and fructose), organic acids (citric acid, malic acid, and propanedioic acid), and amino acids (proline, serine, alanine, phenylalanine, threonine, asparagine, aspartic acid, and glutamic acid). Normalization of peak areas revealed that citric, malic, and propanedioic acids were the most abundant organic acids, consistent with findings in flowers from other species, such as avocado and cocoa. These acids are crucial for metabolism, energy production, and pH balance maintenance during flowering and pollination, thus supporting flower growth and pollen viability (Panchal et al., 2021). Among the amino acids, alanine, aspartic acid, and glutamic acid were predominant. These amino acids, similar to those in corn, cocoa, and avocado flowers, are essential for physiological processes such as pollination, flower development, and metabolic pathways. They are vital for flower health and pollen viability across species (Borghi et al., 2017). Sucrose was the most abundant sugar in durian flowers, consistent with the findings of Bumrungsri et al. (2009), who described sucrose as the predominant sugar during the bud stage, with its levels decreasing after flower opening. This result aligns with studies on flowers in species such as sunflower, corn, and mango, in which sucrose is also reported as the most abundant sugar (Martínez-Noël et al., 2015). Overall, the primary metabolic profiles of durian flowers in this study are consistent with those found in other plant species, highlighting the role of organic acids, amino acids, and sucrose in supporting flower development and pollination. Our findings offer important insights into the biochemical composition of durian flowers and their potential involvement in metabolic pathways related to flowering and pollination.

**Table 1:** Metabolite annotation data from a dual-platform metabolomics approach.

**Group**	**Annotated compound**	**Retention time (min)**	**Adduct**	***m/z***	**Mean of normalized peak area**	**Mass fragment**	**SI**^a^	**Error (mDa)**	**Analytical platform**
Amino acid	L-Alanine, 2TMS derivative	9.32	[M]+	233	0.572 ± 0.02	232, 218, 190, 147, 116, 73, 59	91	-	GC–MS
	L-Proline, 2TMS derivative	13.00	[M]+	259	0.185 ± 0.03	244, 216, 170, 142, 133, 128, 113, 100, 73	89	-	GC–MS
	L-Phenylalanine, 2TMS derivative	13.55	[M]+	309	0.189 ± 0.03	295, 280, 218, 142, 147, 91, 73	85	-	GC–MS
	l-Serine, 3TMS derivative	14.18	[M]+	321	0.222 ± 0.04	306, 278, 218, 204, 188, 163, 147, 116, 100, 73, 59	95	-	GC–MS
	L-Threonine, 3TMS derivative	14.50	[M]+	335	0.156 ± 0.02	320, 294, 276, 230, 218, 191, 159, 147, 129, 117, 101, 86, 73,	92	-	GC–MS
	L-Aspartic acid, 3TMS derivative	16.75	[M]+	349	0.746 ± 0.09	335, 304, 290, 248, 232, 188, 174, 147, 133, 117, 100, 86, 73, 59	94	-	GC–MS
	L-Glutamic acid, 3TMS derivative	18.15	[M]+	363	0.688 ± 0.13	348, 320, 246, 218, 204, 174, 147, 128, 114, 100, 84, 73	87	-	GC–MS
	L-Asparagine, 3TMS derivative	18.84	[M]+	348	0.472 ± 0.24	374, 258, 243, 231, 218, 202, 188, 159, 132, 116, 100, 73, 59	96	-	GC–MS
Sugar	D-Fructose, 5TMS derivative	20.79	[M]+	540	12.020 ± 2.14	361, 319, 257, 230, 217, 204, 191, 157, 147, 117, 103, 73	89	-	GC–MS
	D-Sucrose, 8TMS derivative	29.88	[M]+	918	18.254 ± 1.87	364, 361, 319, 291, 257, 230, 217, 191, 147, 129, 103, 73	89	-	GC–MS
Organic acid	Propanedioic acid, 2TMS derivative	11.30	[M]+	248	0.329 ± 0.08	248, 233, 189, 133, 87, 73	88	-	GC–MS
	Malic acid, 3TMS derivative	14.71	[M]+	350	0.285 ± 0.07	290, 233, 189, 175, 146, 117, 101, 73	85	-	GC–MS
	Citric acid, 4TMS derivative	20.87	[M]+	480	0.945 ± 0.18	374, 365, 347, 333, 319, 305, 285, 273, 257, 230, 183, 147, 73	91	-	GC–MS
	Procyanidin B1	9.47	[M-H]-	578.1991	9.23 ± 1.76	578.1991, 407.0779, 289.0745, 125.3228	–	4.98	UHPLC-ESI-QTOF-MS/MS
	Procyanidin B2	9.65	[M-H]-	579.1474	29.45 ± 1.89	577.1474, 289.0745, 125.3228	–	6.84	UHPLC-ESI-QTOF-MS/MS
Polyphenol	(−)-Epicatechin	9.67	[M-H]-	289.0745	27.46 ± 2.47	289.0745, 245.0829, 203.0735, 151.0413, 109.0285	–	6.24	UHPLC-ESI-QTOF-MS/MS
	Procyanidin C1	9.73	[M-H]-	865.1942	28.69 ± 3.75	865.1942, 577.125, 407.0776, 125.0267	–	8.56	UHPLC-ESI-QTOF-MS/MS
	Quercetin 3-*O*-rhamnoside	12.93	[M-H]-	609.1511	3.98 ± 0.92	609.1511, 300.0292, 151.0004	–	5.72	UHPLC-ESI-QTOF-MS/MS

a percentage similarity was calculated by comparing the obtained mass spectrum with the NIST 20 mass spectrum library.

For the UHPLC–ESI–QTOF–MS/MS analysis, we tentatively identified five secondary metabolites classified as polyphenols, including B1, B2, C1, EP, and quercetin 3-*O*-rhamnoside (Table 1). Based on normalized peak areas, EP and the procyanidin group were annotated as biomarkers for their antioxidant and anti-inflammatory effects. B1, B2, and C1 are classified as B-type procyanidins or condensed tannins, and EP is recognized as a precursor compound of procyanidin. These procyanidins, present in plants such as apples (flesh) and grapes (seeds), are well known for their antioxidant and anti-inflammatory effects (Nie et al., 2023). In addition, quercetin 3-*O*-rhamnoside in durian flowers exhibits antioxidant properties and is involved in the plant's defense mechanisms against pathogens, such as bacteria, fungi, and insects (Pietta, 2000). In addition to cocoa, grapes, and apples, several other flowers contain procyanidins, which contribute to plant defense and provide antioxidant benefits (Yu et al., 2020). These compounds play an essential role in maintaining plant health and may provide potential benefits for human health. In this study, we focused on procyanidins, which are well known for their strong antioxidant and anti-inflammatory properties, and investigated their potential as an alternative source to grape seeds, which are the primary documented source of procyanidins.

### 3.2 Solvent optimization, (−)-epicatechin and procyanidin-containing crude extract production

Binary extraction systems are more effective than single-component systems. In particular, ethanol–water mixtures improve the polyphenol extraction efficiency from crop wastes such as grape pomace, mango seed kernels, and early immature durian fruit (Pewlong et al., 2025). In this study, we varied the ethanol concentrations to determine the most effective extraction solvent by measuring the antioxidant activity and total phenolic content. We selected ethanol because it allows for the safer extraction of valuable compounds than methanol, and its production process is more environmentally friendly. Looking forward, if the process is scaled up to industrial level, bioethanol could be used with the added potential for solvent recycling. Finally, any remaining cell residues can be dried and converted into biochar or used as animal feed, thus supporting the concept of a zero-waste approach (Potijun et al., 2021). Although freeze-drying increases production costs, our previous research on procyanidins and epicatechin in early immature durian found that using heat or air-drying resulted in a reduction of over 60 % in compound levels (Pewlong et al., 2025).

**Fig. 2:**
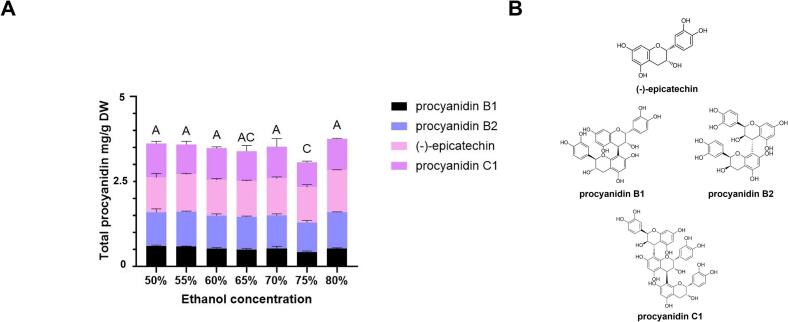
Procyanidin content. (A) Total procyanidin content (mg/g DW) in durian flower extracts obtained using different ethanol concentrations (% v/v) (including (−)-epicatechin)). Data are presented as the mean ± standard deviation (SD). Statistical analysis was performed using one-way ANOVA with 95 % confidence intervals (*p* < 0.05). Significant differences between groups are indicated by different letters (A, B, and C). (B) Chemical structure of procyanidins in durian flower extracts.

We performed HPLC analysis to quantify the extracted compounds. Our results indicate that 80 % ethanol is the most effective solvent (Fig. 2A and B), consistent with previous studies using 50 %–80 % ethanol concentrations for extracting polyphenol-rich compounds.

To assess the biological activity of the extract, chemical analyses were conducted, including FRAP and FC assays. Our results revealed no significant differences in total phenolic content and antioxidant activity across ethanol concentrations ranging from 50 % to 80 % (SFig. 1). Therefore, we selected 80 % ethanol for further extractions, as it yielded the highest (−)-epicatechin and procyanidin content.

**Fig. 3:**
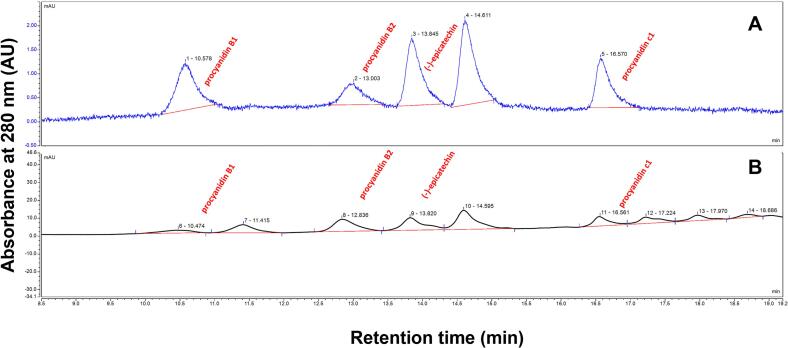
HPLC chromatogram of (−)-epicathechin and procyanidins at A_280_. (A) Authentic standard compounds: procyanidin B1, procyanidin B2, (−)-epicatechin, and procyanidin C1 (6.25 μg/mL). (B) Durian flower extract at 50 mg/mL.

Upon HPLC analysis, we identified and quantified procyanidin compounds in the crude extract, including B1, B2, C1, and EP. We obtained quantitative results by comparing retention times (Fig. 3) and establishing a linear relationship with authentic standards (SFig. 2). The limits of detection (LOD) and quantification (LOQ) are shown in Table S3. Following solvent evaporation, we obtained a crude extract yield of 30.67 % (based on dried flower weight). The B1, B2, C1, and EP concentrations in the crude extract of durian flower were 1.08, 2.10, 2.13, and 2.37 mg/g, respectively, resulting in a total procyanidin content of 7.68 mg/g (Table 2).

**Table 2:** (−)-Epicatechin and procyanidin contents in durian flower crude extract (mg/g). All values are represented as the means ± SD of biological triplicates.

Compound	Concentration (mg/g)
Procyanidin B1	1.08 ± 0.37
Procyanidin B2	2.10 ± 0.77
Procyanidin C1	2.13 ± 0.46
(−)-Epicatechin	2.37 ± 0.31

The profiles of the identified procyanidin compounds in durian flowers were similar to those found in early immature durian (Pewlong et al., 2025) and grapes (Rodríguez-Pérez et al., 2019). EP, a monomer with anti-inflammatory effects, lowers blood sugar levels and acts as an antioxidant. In contrast, dimer compounds (such as B1 and B2) exhibited higher antioxidant activity than EP. Procyanidins can inhibit cancer cell growth by modulating the PI3K/Akt and JNK pathways, thereby promoting apoptosis (Lee Y., 2017). In addition, the trimer PC1 prevents UVR-induced skin damage and collagen degradation (Katiya, 2016). Overall, durian flower crude extracts exhibit a high total procyanidin content, indicating their potential for significant antioxidant and anti-inflammatory effects.

Moreover, durian flowers, which are typically considered agricultural waste, can be used as an alternative source of procyanidin. The total procyanidin content in durian flowers was comparable to that of well-known sources, such as cocoa, apples, and grapes. In cocoa, the procyanidin concentration ranged from 10 to 20 mg/g dry weight (DW) in crude extracts, depending on the extraction method (Papillo et al., 2019). In apples, particularly in flavonoid-rich peel extracts, procyanidin concentrations ranged from 12 to 15 mg/g DW (Chung et al., 2017). Grape seeds, which are rich in polyphenol compounds, can yield crude extracts with procyanidin concentrations of approximately 30 mg/g DW (Unusan, 2020). Based on these results, durian flowers appear to be an efficient alternative source for procyanidin production.

### 3.3 Cytotoxicity of durian flower crude extract

Next, we evaluated the cytotoxicity of durian flower crude extract for potential cosmeceutical and nutraceutical applications using the MTT cell viability assay to monitor cell proliferation. Treatment with the crude extract induced dose-dependent cell death over a 24-h period (SFig. 3). At lower concentrations (below 125 μg/mL), we detected no significant cell death. However, we observed a marked reduction in cell viability at concentrations from 500 μg/mL, similar to the results from our previous study on early immature durian (Pewlong et al., 2025). We determined that the toxic dose was 1211.07 μg/mL, which caused 50 % cell death (Table 3). In accordance with the OECD and ISO guidelines, we selected concentrations of 125 and 250 μg/mL for subsequent studies to ensure that cell viability remained above 70 %, as such concentrations are considered non-cytotoxic or nonirritant and are widely accepted for *in vitro* safety testing. Consequently, we conclude that durian flower extract is safe for use as a cosmeceutical ingredient and can be used for future nutraceutical development.

**Table 3:** Cell viability and IC_50_ values of the durian flower crude extract treatment on HaCaT cells.

Concentration (μg/mL)	Viability (%)	IC_50_
2000	20.76 ± 2.30	1211.07 ± 122.19 (μg/mL)
1000	57.69 ± 7.58	
500	68.17 ± 6.32	
250	85.27 ± 7.61	
125	102.73 ± 8.43	
62.5	103.83 ± 8.65	
31.25	102.72 ± 7.92	
0 (control)	99.99 ± 0.02	

The data represent the percentage of cell viability. IC_50_ corresponds to the concentration that inhibited 50 % of cell viability. All values are represented as the means ± SD of biological triplicates.

### 3.4 Protective effects of durian flower crude extract against UVA-induced oxidative stress and inflammation in HaCaT cells

UVA radiation plays a significant role in skin aging and DNA damage, particularly in the epidermis, which is the layer most exposed to sunlight. Prolonged UVA exposure triggers free-radical production within cells, leading to premature aging, reduced skin strength, and increased risk of skin cancer development (Amaro-Ortiz et al., 2014; Narayanan et al., 2010). UVA radiation is absorbed by skin chromophores, which trigger the generation of oxidative stress through ROS and activate transcription factors such as NF-κB and AP-1. This activation promotes the release of inflammatory cytokines, including TNF-α and IL-6. which contribute to skin inflammation, cellular damage, and extracellular matrix degradation, ultimately resulting in skin aging and damage (Kammeyer & Luiten, 2015).

**Fig. 4:**
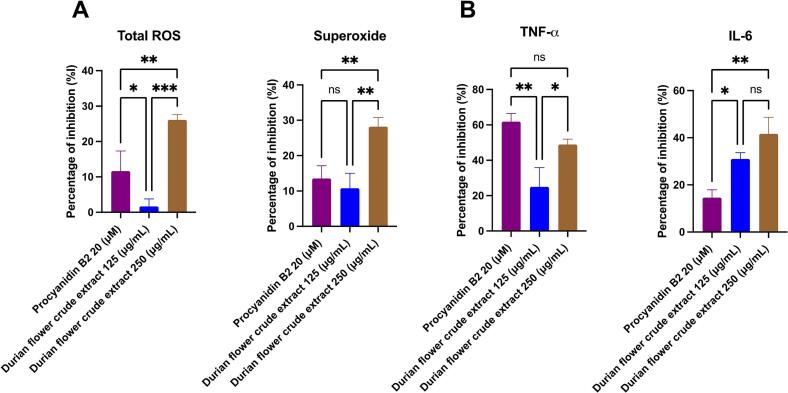
Inhibitory effects of procyanidin B2 and the crude extract of durian flowers on reactive oxygen species (ROS) and pro-inflammatory cytokines. (A) Percent inhibition of total ROS and superoxide production. (B) Percent inhibition of TNF-α and IL-6 secretion. Data are expressed as mean ± standard deviation (SD) from three independent experiments. Statistical significance was determined using ANOVA followed by post hoc analysis (*p < 0.05, **p < 0.01, ***p < 0.001; ns = not significant).

In this study, we evaluated the antioxidant activity of durian flower crude extract by exposing HaCaT cells to UVA radiation at a dose of 7 J/cm^2^. Previous studies on human keratinocytes from breast tissue demonstrated that UVA exposure at 6.75 J/cm^2^ and 12.5 J/cm^2^ induced comparable P53 mutations, indicating no significant increase in mutation rates beyond 6.75 J/cm^2^ (Huang, Berner, & Halliday, 2009). We monitored the inhibition of two free-radical types (i.e., ROS and superoxide). Our results indicated that the crude extract of durian flowers effectively inhibited free radicals at a concentration of 250 μg/mL, with inhibition rates of approximately 26.1 % and 28.2 % for total ROS and superoxide, respectively (Fig. 4A and Table S2). In contrast, the lower concentration of 125 μg/mL yielded only minimal inhibitory effects. Durian flower extract at 125 μg/mL exhibited a comparable level of free-radical inhibition as procyanidin B2, the positive control. Our results closely align with those of a previous study on procyanidins in early immature durian fruit (Pewlong et al., 2025), in which procyanidin B2 exhibited the highest biological activity. Similarly, in grapes, procyanidin B2 is the predominant form and is well-documented for its prominent anti-inflammatory and antioxidant properties (Rodríguez-Pérez et al., 2009). Our experimental results confirmed that the crude extract of durian flower effectively inhibits ROS at a concentration of 250 μg/mL in HaCaT cells, which are keratinocytes that are directly exposed to UVA radiation.

In addition, UVA exposure induces an inflammatory response in skin cells, representing a key event in skin vulnerability. To investigate inflammation inhibition, an experiment was performed to assess the suppression of TNF-α and IL-6 (Wroński et al., 2024). UVA exposure induces DNA damage, cellular inflammation, and matrix degradation. In response to inflammation, cells release various cytokines to communicate and stimulate immune responses, with TNF-α being the first cytokine secreted during acute inflammation. This, in turn, triggers the release of other interleukins, particularly IL-6, playing a crucial role in keratinocyte proliferation, particularly during wound healing or injury (Piipponen and Li, 2020). Our experiment demonstrated that crude extract from durian flowers effectively inhibited inflammation in HaCaT cells. Notably, at a concentration of 250 μg/mL, the extract reduced UVA-induced TNF-α and IL-6 levels by 48.9 % and 41.6 %, respectively (Fig. 4B and Table S2).

Previous studies have assessed the role of (−)-epigallocatechin-3-gallate from green tea in providing skin photoprotection against UVR. This compound acts as a potent antioxidant, scavenging ROS and protecting against UVR-induced inflammation, wrinkling, and aging by modulating the NF-κB and MAPK pathways (Katiyar et al., 2001). Therefore, application of procyanidin-rich crude extract of durian flowers at a concentration of 250 μg/mL can significantly reduce oxidative stress, an important contributor to free-radical production in skin cells. This effect can help prevent inflammatory processes that could otherwise compromise skin immunity and increase the risk of developing skin cancer. With the anti-inflammatory and antioxidant activities of procyanidins, particularly in reducing oxidative stress and inflammation in humans exposed to various abiotic stressors such as UVR and H_2_O_2_ (Katiyar, 2016), this extract could efficiently contribute to mitigating the effect of key stressors. Climate change–related stressors, such as PM2.5 and PM10, are rapidly emerging global concerns, potentially contributing to various skin conditions, including dermatitis, chronic psoriasis, acne, and premature skin aging, which are increasingly prevalent worldwide (Paik et al., 2024).

Furthermore, the extract that we investigated can reduce skin aging and prevent wrinkle formation. Moreover, it is nontoxic to skin cells and has immediate potential for cosmeceutical use. As a sustainable and cost-effective alternative to grape-derived procyanidins, it also contributes to reducing agricultural waste from durian cultivation. This positions it as a promising candidate for future nutraceutical product development, not only because of its bioactivities but also as a functional ingredient that can enhance the efficacy of health supplements.

## 4 Conclusions

Durian flowers, which are often discarded during the flowering stage to optimize fruit production, represent an untapped resource of bioactive compounds. In this study, using dual-platform metabolomic analysis, we annotate key polyphenolic compounds, particularly (−)-epicatechin and procyanidins. HPLC analysis quantified the total crude procyanidin content (including (−)-epicatechin) at 7.68 mg/g, with notable compounds including (−)-epicatechin and procyanidins B1, B2, and C1, all of which are recognized for their potent anti-inflammatory and antioxidant activities. We conducted *in vitro* studies exposing HaCaT cells to UVA-induced oxidative stress, demonstrating that crude extracts of durian flower significantly reduced total ROS and superoxide levels by 28 %. Additionally, the crude extracts reduced the levels of pro-inflammatory markers, lowering TNF-α and IL-6 levels by 48.9 % and 41.6 %, respectively. Our findings indicate that durian flower extract, which is rich in procyanidins, exhibits significant anti-inflammatory and antioxidant activities against UVA-stimulated skin cells. These findings highlight the potential of durian flower extracts for immediate application in the cosmeceutical industry. Furthermore, durian flowers are an abundant agricultural byproduct in Southeast Asia, helping to alleviate concerns regarding the cost of procuring raw materials.

## CRediT authorship contribution statement

**Supakorn Potijun:** Writing – review & editing, Writing – original draft, Visualization, Validation, Software, Methodology, Investigation, Formal analysis, Conceptualization. **Nattaya Pattarapipatkul:** Methodology. **Pitchakorn Boonma:** Methodology. **Putthamas Pewlong:** Methodology. **Intira Pathtubtim:** Methodology. **Thanchanok Muangman:** Methodology. **Bunyarit Meksiriporn:** Writing – review & editing. **Hubert Schaller:** Writing – review & editing, Supervision. **Supaart Sirikantaramas:** Writing – review & editing, Supervision, Project administration, Funding acquisition, Conceptualization.

## Funding

This study received financial support from the Thailand Science Research and 10.13039/100012774Innovation Fund, 10.13039/501100002873Chulalongkorn University (FF_68_312_2300_032) (to S.S.) and the 90^th^ Anniversary of 10.13039/501100002873Chulalongkorn University Scholarship under the Ratchadaphisek Somphot Endowment Fund (to S.P. and S.S.). Additionally, S.S. and H.S. received funding from the Franco-Thai Mobility Programme/PHC SIAM 2025–2026 for international exchange. The funding organizations had no involvement in the study's design, sample collection, data analysis, interpretation, or manuscript preparation.

## Declaration of competing interest

The authors declare that they have no known competing financial interests or personal relationships that could have appeared to influence the work reported in this paper.

## Data Availability

Data will be made available on request.

## Appendix A Supplementary data

Supplementary material. Image 1
