# Supplementary material for: Insights from dual-platform metabolomics on durian flowers: An alternative source of procyanidins from agricultural waste with bioactivities
Source: Food Chem X. 2025 Jul 1;29:102719. doi: 10.1016/j.fochx.2025.102719 (PMC12275947; doi:10.1016/j.fochx.2025.102719)

**Supplementary data**

**Insights from Dual-Platform Metabolomics on Durian Flowers: An Alternative Source of Procyanidins from Agricultural Waste with Bioactivities**

Supakorn Potijun, Nattaya Pattarapipatkul, Pitchakorn Boonma, Putthamas Pewlong, Intira Pathtubtim, Thanchanok Muangman, Bunyarit Meksiriporn, Hubert Schaller, and Supaart Sirikantaramas

**Table S1**

Retention index used for primary metabolite annotation in GC-MS analysis^1^.

| **Group** | **Annotated compound** | **Calculated retention index** | **Database retention index (NIST 18)** | **Chemical structure** |
| --- | --- | --- | --- | --- |
| Amino acid | L-Alanine, 2TMS derivative | 1068 | 1038 | 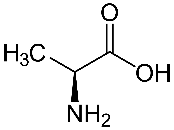 |
|  | L-Proline, 2TMS derivative | 1356 | 1258 | 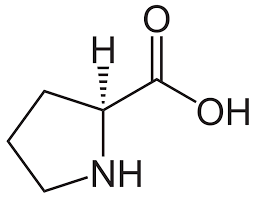 |
|  | L-Serine, 3TMS derivative | 1472 | 1322 | 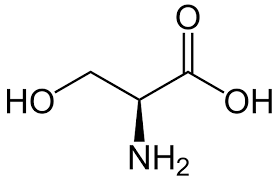 |
|  | L-Threonine, 3TMS derivative | 1444 | 1357 | 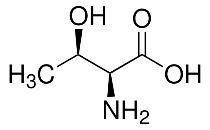 |
|  | L-Aspartic acid, 3TMS derivative | 1671 | 1512 | 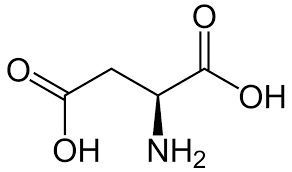 |
|  | L-Glutamic acid, 3TMS derivative | 1852 | 1612 | 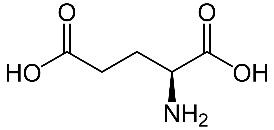 |
|  | L-Phenylalanine, 2TMS derivative | 1853 | 1711 | 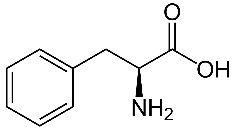 |
|  | L-Asparagine, 3TMS derivative | 1855 | 1745 | 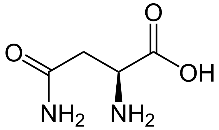 |
| Sugar | D-Fructose, 5TMS derivative | 2097 | 1982 | 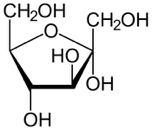 |
|  | D-Sucrose, 8TMS derivative | 3185 | 3552 | 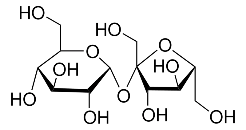 |
| Organic acid | Propanedioic acid, 2TMS derivative | 1193 | 1070 | 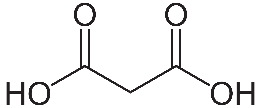 |
|  | Malic acid, 3TMS derivative | 1521 | 1390 | 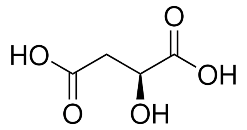 |
|  | Citric acid, 4TMS derivative | 2183 | 1944 | 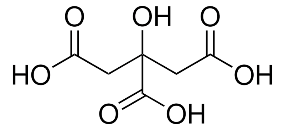 |

^1^ Metabolites were **annotated** by comparing their mass spectra and retention indexes (RIs) to entries in the NIST 18 database. While experimental RIs are presented alongside database values, minor variations may occur due to differences in chromatographic conditions and matrix effects. This supports the preliminary nature of these annotations.

| Sample | Percentage of inhibition (%I) | | | |
| --- | --- | --- | --- | --- |
|  | Antioxidation | | Anti-inflammation | |
|  | Total ROS | Superoxide | TNF-α | IL-6 |
| Procyanidin B2 20 (μM) | 11.649±5.663 | 13.546±3.616 | 61.855±4.627 | 14.567±3.365 |
| Durian flower crude extract 125 (μg/mL) | 2.633±1.867 | 10.800±4.228 | 24.938±10.899 | 31.048±2.613 |
| Durian flower crude extract 250 (μg/mL) | 26.104±1.500 | 28.172±2.617 | 48.913±2.960 | 41.606±5.986 |

**Table S2.** Inhibition of antioxidant and anti-inflammatory activities (procyanaidin B2 as the positive control). All values are presented as means ± SD of three biological replicates.

**Table S3.** Summary of slopes, SD, LOD, and LOQ for procyanidins B1, procyanidins B2, (−)-epicatechin, and procyanidins C1. Data reflects the linear regression and sensitivity of the HPLC method for each authentic compound.

| Compounds | Slope | SD | LOD (μg/mL) | LOQ (μg/mL) |
| --- | --- | --- | --- | --- |
| Procyanidin B1 | 0.0140 | 0.00347 | 0.82 | 2.48 |
| Procyanidin B2 | 0.0143 | 0.00636 | 1.47 | 4.45 |
| (−)-epicatechin | 0.0177 | 0.00516 | 0.96 | 2.92 |
| Procyanidin C1 | 0.0099 | 0.00333 | 1.11 | 3.36 |

**Figure S1** Total phenolic content and antioxidant properties of durian flower extracts the total phenolic content is expressed as mg of gallic acid/g of early immature durian flower while, the FRAP-reducing capacity is expressed as mM Fe²⁺/mg of durian flower. All values are presented as means ± SD of three biological replicates, different letters with subscripts indicate significant differences (P < 0.05).

*^
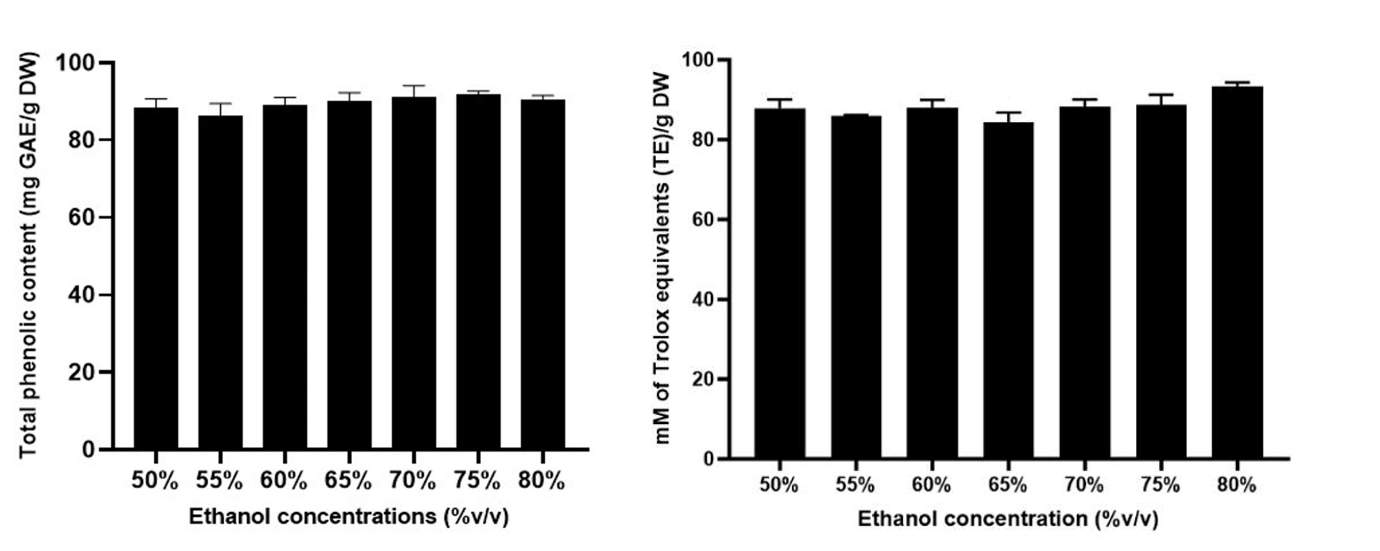
^*

**Figure S2** Equation, R^2^, and regression line of major parent compounds commercial authentic standard.


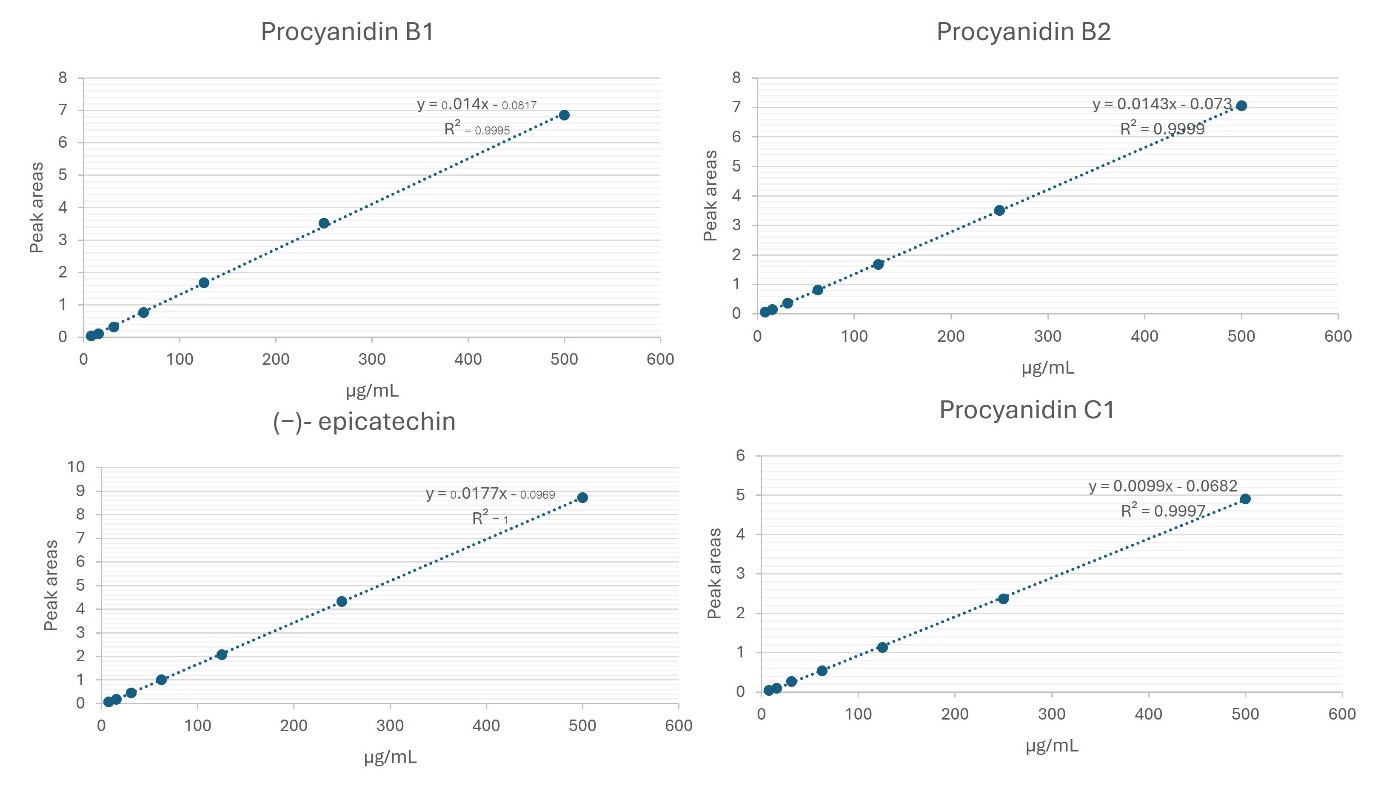


**Figure S3** Cytotoxic effects of durian flower crude extract on HaCaT cells (Passaged at 25). Cell viability was assessed using an MTT assay after 24 hours of treatment with varying concentrations of the extract. Data are expressed as mean ± standard deviation (SD) from three independent experiments.


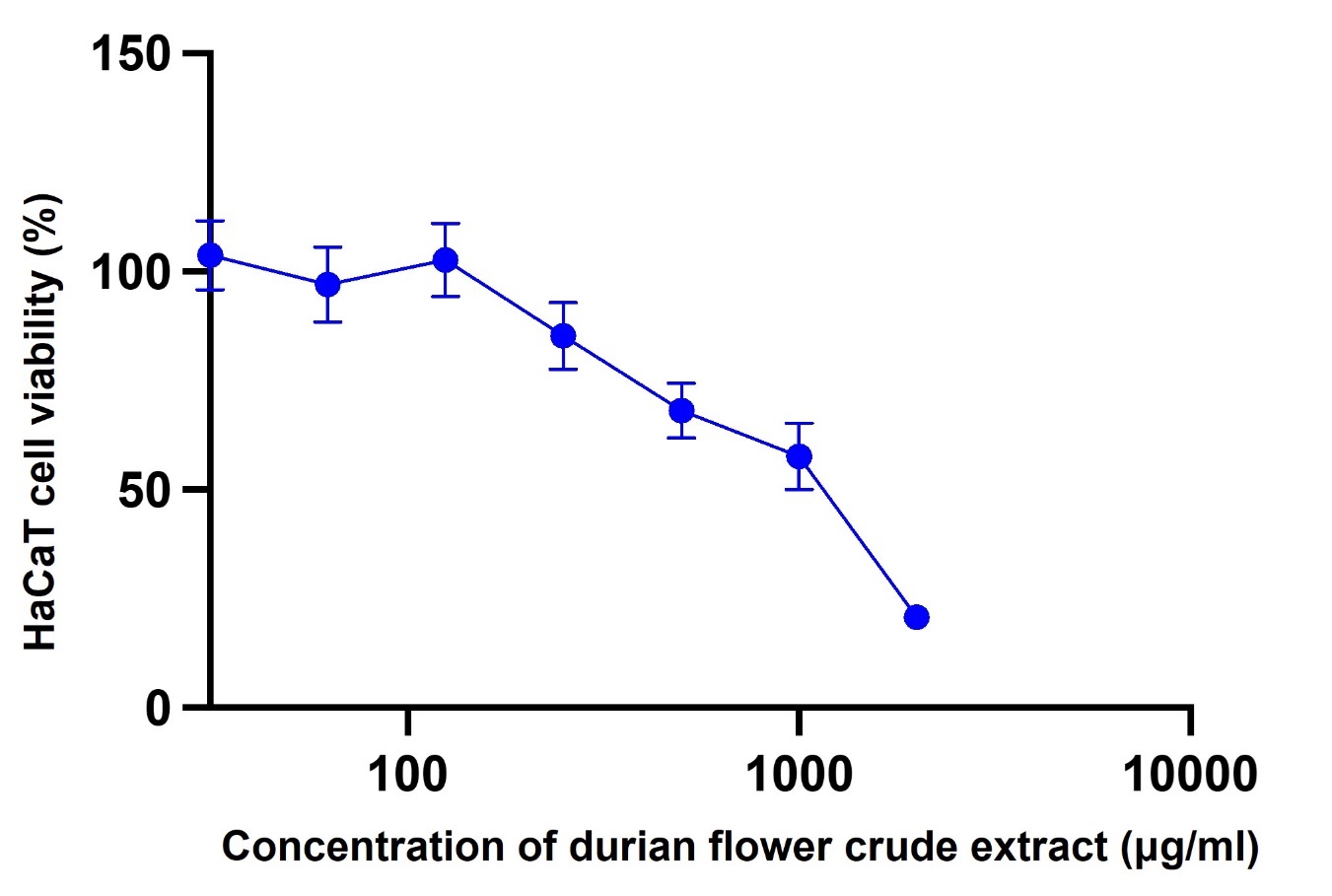

Supplement: Supplementary file 1 — Supplementary material. [file mmc1.docx]
